# Lifestyle-related risk factors and recurrence after ventral hernia surgery: nationwide registry and survey study

**DOI:** 10.1093/bjsopen/zrag098

**Published:** 2026-08-11

**Authors:** Sofie Anne-Marie Skovbo Jensen, Siv Fonnes, Anders Gram-Hanssen, Hanne Tønnesen, Hugin Reistrup, Jacob Rosenberg, Susanne Vahr Lauridsen, Sara Nørgård Jensen, Jason Joe Baker

**Affiliations:** Center for Perioperative Optimization, Department of Surgery, Copenhagen University Hospital—Herlev and Gentofte, Herlev, Denmark; World Health Organization Collaborating Centre, The Parker Institute, Bispebjerg and Frederiksberg Hospital, Frederiksberg, Denmark; Center for Perioperative Optimization, Department of Surgery, Copenhagen University Hospital—Herlev and Gentofte, Herlev, Denmark; Center for Perioperative Optimization, Department of Surgery, Copenhagen University Hospital—Herlev and Gentofte, Herlev, Denmark; World Health Organization Collaborating Centre, The Parker Institute, Bispebjerg and Frederiksberg Hospital, Frederiksberg, Denmark; Department of Health Sciences, Faculty of Medicine, Lund University, Lund, Sweden; Center for Perioperative Optimization, Department of Surgery, Copenhagen University Hospital—Herlev and Gentofte, Herlev, Denmark; Center for Perioperative Optimization, Department of Surgery, Copenhagen University Hospital—Herlev and Gentofte, Herlev, Denmark; Department of Clinical Medicine, University of Copenhagen, Copenhagen, Denmark; Center for Perioperative Optimization, Department of Surgery, Copenhagen University Hospital—Herlev and Gentofte, Herlev, Denmark; World Health Organization Collaborating Centre, The Parker Institute, Bispebjerg and Frederiksberg Hospital, Frederiksberg, Denmark; Department of Clinical Medicine, University of Copenhagen, Copenhagen, Denmark; Department of Statistics, Sanos, Søborg, Denmark; Center for Perioperative Optimization, Department of Surgery, Copenhagen University Hospital—Herlev and Gentofte, Herlev, Denmark

## Abstract

**Background:**

The study evaluated the associations of individual and combined lifestyle-related risk factors with long-term recurrence after primary and incisional ventral hernia repair compared with patients without lifestyle-related risk factors.

**Methods:**

This nationwide study used registry and survey data from Danish patients who underwent ventral hernia repair between 2014 and 2024. Patients were identified in the Danish National Patient Register, after which digital surveys were distributed. Survey data on recurrence and lifestyle factors were linked with data from the Danish Ventral Hernia Database and Danish National Patient Register. The outcome was hernia recurrence (reoperation or self-reported). Average treatment effects were estimated using adjusted logistic regression and standardization (G-computation).

**Results:**

In total, 18 959 patients were included in the study, comprising 15 186 patients undergoing primary ventral repair and 3773 undergoing incisional hernia repair (response rate 78%). More than half the patients reported one or more lifestyle-related risk factors. For primary ventral hernia repair, the adjusted population-averaged recurrence prevalence was 14% without lifestyle-related risk factors. Smoking, obesity, and their combination were associated with a higher recurrence prevalence, with absolute differences of 3% (95% confidence interval (c.i.) 0% to 5%), 3% (95% c.i. 2% to 5%), and 6% (95% c.i. 2% to 10%), respectively. Other lifestyle combinations showed similar trends but were less consistently significant across analyses. For incisional hernia repair, the adjusted population-averaged recurrence prevalence was 25% without lifestyle-related risk factors. Smoking and obesity were associated with absolute increases in recurrence prevalence of 6% (95% c.i. 0% to 11%) and 5% (95% c.i. 1% to 9%), respectively.

**Conclusions:**

Smoking and obesity were associated with a higher recurrence prevalence after primary ventral and incisional hernia repair. Associations for combined lifestyle factors were less consistent overall. Given the number of analyses performed, the findings should be interpreted with caution.

## Teaser

In this nationwide study of 18 959 Danish patients with ventral hernia repair, more than half the patients reported at least one lifestyle risk factor. Smoking, obesity, and their combination were associated with a 3–6% higher prevalence of recurrence after primary ventral hernia repair compared with recurrence prevalence among those with no lifestyle risk factors. For incisional hernias, smoking and obesity were associated with a 5–6% higher recurrence prevalence.

## Introduction

Ventral hernias are known to often negatively affect patients’ quality of life^1,2^, and every year many patients undergo ventral hernia repair^3^. Recurrence estimates vary widely across the literature, ranging from a few per cent to as high as around 70%^4,5^, and generally increase with the duration of follow-up^5^. Recurrence depends on many factors, such as the type of hernia, defect width, the use (or not) of mesh, and complications like surgical site infections and reoperation^5,6^. However, in recent decades, lifestyle-related risk factors, such as smoking, obesity, high alcohol intake, and physical inactivity, have become increasingly recognized as modifiable risk factors for short-term postoperative complications, both across surgical procedures generally^7–10^ and in ventral hernia surgery specifically^11^. Smoking, obesity, and high alcohol intake are known risk factors for surgical site infections in general^12–14^, and in ventral hernia repair, such surgical site infections increase the risk of recurrence^6,15^. A higher frequency of preoperative exercise has been associated with fewer short-term complications after ventral hernia repair^16^. However, the effect of preoperative exercise on long-term outcomes, such as recurrence, is unknown. Overall, the impacts and magnitude of these four individual lifestyle-related risk factors (smoking, obesity, high alcohol intake, and exercise) on recurrence after ventral hernia surgery are not well established^17,18^, and evidence regarding co-existing factors is even more sparse.

This study hypothesized that the prevalence of recurrence after ventral hernia repair would be higher among patients with lifestyle-related risk factors, and that an increasing number of lifestyle-related risk factors would correlate with a higher prevalence of hernia recurrence. The primary objective of the study was to evaluate the association of individual and combined lifestyle-related risk factors with recurrence after primary ventral and incisional hernia repair, respectively, compared with patients with no lifestyle-related risk factors.

## Methods

This nationwide study was based on register and survey data from patients who had undergone ventral hernia repair in Denmark between 1 January 2014 and 31 March 2024. The study was part of the AFTERHERNIA Project^19^ and is reported according to the RECORD^20^ and CROSS^21^ guidelines. Analyses were prespecified in a statistical analysis plan and uploaded to an open repository^22^.

In the AFTERHERNIA Project, patients were identified from the Danish National Patient Register^23^, which is a comprehensive mandatory nationwide register with routinely collected person-level information on all private and public hospital contacts in Denmark. Patients were identified based on the Nordic Medico-Statistical Committee classification of surgical procedure codes that indicated a hernia surgery^24^. In the event that patients had undergone surgery for multiple ventral hernias during the study period, they were only included once, based on their most recent ventral hernia surgery. The mandatory personal identification number that all Danish residents are assigned to is used in all contacts with both private and public healthcare services and in all official registries^25^. This ensured that person-level data could be linked to the Danish Civil Registration System^25^, providing live data on whether patients were deceased or had emigrated. The unique personal identification number is also linked to a mandatory digital personal communication platform, Digital Post, which was used to distribute the surveys. Digital Post is available to residents in Denmark and used by 95% of residents aged ≥ 15 years^26^.

The AFTERHERNIA Project sent a survey to all patients identified as described above, excluding those who were dead, emigrated, exempt from Digital Post, or had a protected street address that prevents marketing and uninvited contact. The survey was distributed between August 2024 and January 2025. REDCap^27^ was used for data collection and to create unique personal links that could only be responded to once. Reminders were sent three times at weekly intervals, whereafter non-responders were contacted by telephone once. Patients were excluded if they reported having insufficient Danish skills or mental or physical impairment prohibiting them from completing the questionnaire. The survey included questions about hernia recurrence and current lifestyle-related risk factors^19^, which were used in this study. Patients were asked whether they suspected that their hernia had reoccurred and whether this had been clinically confirmed by a medical doctor. They were also asked about their lifestyle at the time of the survey, including smoking status, height and weight, weekly alcohol intake, and level of weekly physical activity. Physical activity was defined as ≥ 3.5 h/week and was assessed with a single yes/no item, with examples across domains (leisure, transport, occupational, and household activities) to standardize interpretation. Although obesity is not a lifestyle behaviour *per se*, it was analysed alongside the other lifestyle-related risk factors because it represents a potentially modifiable factor associated with surgical outcomes. Survey data were then linked to perioperative and demographic data from the Danish Ventral Hernia Database^28^. Surgeons in both private and public hospitals are obliged to prospectively register perioperative data on all ventral hernia surgeries immediately after each surgery. The registration rate in 2024 was 82%^29^, and the database has been validated against medical records, with 89–99% accuracy^30^.

Inclusion and exclusion criteria were applied to the data set, consisting of data from the linked registries and the survey. Patients eligible for inclusion in the study were those aged ≥ 18 years who had undergone elective primary ventral hernia repair, defined as umbilical or epigastric, or incisional hernia repair. Exclusion criteria were a hernia defect width > 10 cm, other hernias, emergency surgery, first registered repair in the Danish Ventral Hernia Database being a repair for recurrence, missing data on lifestyle-related risk factors from the survey, and index surgery not registered in the Danish Ventral Hernia Database, because information on potential confounders, such as hernia defect width, would be missing. The response rate was calculated as the proportion of distributed questionnaires eligible for inclusion in this study that were fully completed, excluding patients who had indicated that they did not want to participate.

**Figure 1:**
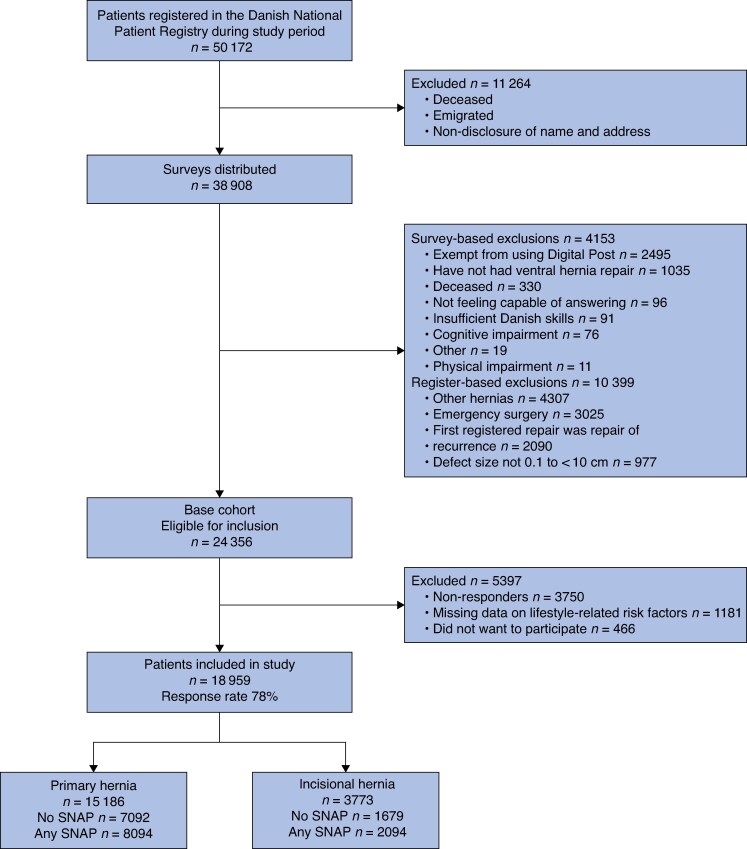
Flow chart of data selection and collection All patients included in the study were also included in the main analyses. Digital Post is a mandatory digital personal communication platform available to residents in Denmark that was used to distribute the surveys. SNAP: smoking (daily), nutrition (BMI > 30 kg/m^2^), high alcohol intake (> 10 units/week), and physical inactivity (defined as physical activity < 3.5 h/week).

The sample consisted of all adult patients undergoing ventral hernia repair in the study period who had answered the survey and fulfilled the eligibility criteria (*Fig. 1*). Follow-up was defined as the time from index surgery (that is, the first registered primary ventral or incisional hernia repair in the study period) to reoperation for recurrence or answering the survey.

### Data collection and variables

The primary outcome in this study was recurrence, defined as either self-reported recurrence from the survey or register-based reoperation. Reoperation was defined as a new registration of a surgical procedure code for the same type of hernia (umbilical, epigastric, or incisional) as the index procedure during the study period. Data from the Danish Ventral Hernia Database were provided by the Danish Healthcare Quality Institute, which also receives data from the Danish National Patient Register. This ensured that any reoperations not recorded in the Hernia Database were still captured through the National Patient Register. Variables of interest were postoperative self-reported lifestyle-related factors in the survey: smoking status (never, ex-smoker, and current daily smoker); alcohol intake (categorized as the intake of 0–10, 11–20, and > 20 units/week); height in centimetres and weight in kilograms (to calculate body mass index (BMI)); and being physically active > 30 min/day or 3.5 h/week (yes/no). Data from the Danish Ventral Hernia Database were perioperative details from the index surgery, including the date, type of hernia, hernia defect width, mesh use and placement, and surgical approach (open, laparoscopic, or robot-assisted), and patient characteristics. Finally, marital status was recorded in the Danish Civil Registration System, and the Charlson co-morbidity index was calculated based on diagnoses registered at any hospital in Denmark from the Danish National Patient Register.

### Statistical analysis

The effect of lifestyle-related risk factors (smoking, BMI, alcohol intake, and physical inactivity) on the prevalence of ventral hernia recurrence was estimated as average treatment effects using adjusted logistic regression with standardization (G-computation) to control for confounding. In this way, marginal prevalence values for each lifestyle-related risk factor individually and for their combinations were estimated as if all groups had the same distribution of covariates. The logistic regression models were constructed to adjust for the confounders of age, sex, width of hernia defect, mesh *versus* non-mesh, Charlson co-morbidity index (five categories), surgical approach, and time since surgery. Results are presented as marginal (that is, population-averaged) prevalences and/or prevalence differences with their 95% confidence interval (c.i.) and *P* values. Two main analyses were conducted. In one analysis, lifestyle-related risk factors were dichotomized and included as one categorical variable with all 16 possible combinations of the four dichotomized lifestyle factors. Associations with recurrence were estimated for all combinations. The reference group comprised patients without lifestyle-related risk factors, defined as never or previous smokers, BMI < 30 kg/m^2^, alcohol intake of 0–10 units/week, and being physically active. Due to the low reliability of results in groups with few patients, estimates relying on less than 50 patients are not shown. The analysis was also performed for subgroups according to the size of the hernia defect width (> 4 *versus* ≤ 4 cm) and surgical approach (open *versus* laparoscopic and robotic), and a sensitivity analysis was conducted with follow-up < 5 years and one restricting the outcome to clinically confirmed recurrences (reoperation or clinically confirmed). In the second analysis, associations with each lifestyle factor were estimated separately, using more detailed categorical levels and adjusting for the other lifestyle factors. Here, the reference groups were defined as never-smokers, BMI 18–25 kg/m^2^, alcohol intake 0–10 units/week, and being physically active to allow for more nuanced comparisons within each factor across different exposure levels. All analyses were based on complete cases without imputations, and statistical significance was defined as two-sided *P* < 0.05. All data cleaning and analyses were conducted in R® version 4.5.1 (R Foundation for Statistical Computing, Vienna, Austria), and standardization was performed using the Targeted package.

Assessing the agreement between data from the time of surgery (registry-based) and current self-reported survey data for smoking status and BMI was planned with the use of McNemar’s test and a paired *t* test, respectively. In addition, within-person changes in BMI were visualised using histograms, and the proportion of patients with changes in BMI exceeding ±5 kg/m^2^ was calculated. However, the test for smoking status was found to be of limited value because differences in response options and data inconsistencies between the Danish Ventral Hernia Database^28^ and the AFTERHERNIA survey reduced the interpretability of the results.

Patients with ventral hernia and incisional hernia repairs were analysed separately in all analyses^31^. All statistical analyses were planned in collaboration with and performed by a statistician (S.N.J.).

### Ethical considerations

The study was approved by the Danish Data Protection Agency (p-2023-14805) and was registered with the Danish Health Data Authority (FSEID-00006834). According to Danish law, no ethics approval was necessary. Informed consent was collected from all patients when they were answering the survey.

## Results

**Figure 2:**
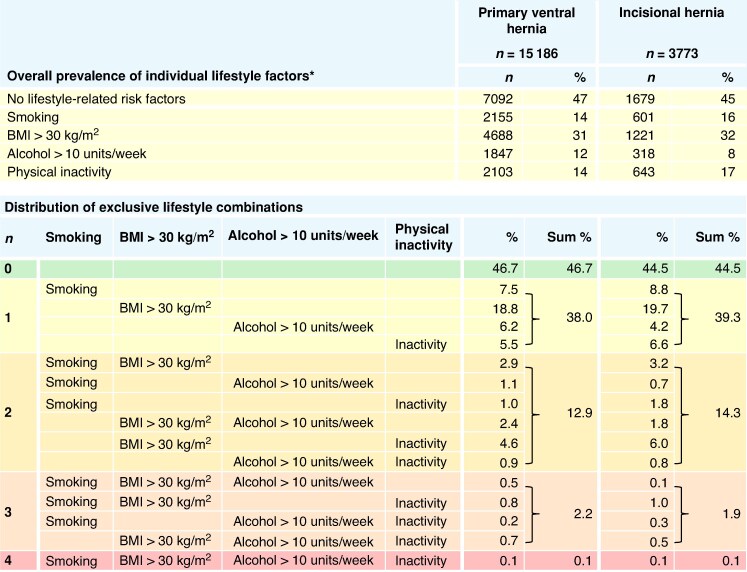
Distribution of lifestyle-related risk factors in the ventral hernia population The top panel shows the overall prevalence of each lifestyle-related risk factor in the two hernia populations. The bottom panel shows the exclusive distribution of lifestyle factors and their specific combinations. Smoking is defined as daily smoking. Physical inactivity is defined as < 3.5 h of physical activity per week. *Because some patients reported more than one lifestyle-related risk factor, individuals may be represented in multiple categories; therefore, the sum of counts and percentages exceeds the total number of patients. BMI, body mass index.

The study included 18 959 patients, of whom 15 186 had undergone primary ventral hernia repair and 3773 had undergone incisional hernia repair, with a total response rate of 78% (*Fig. 1*). In both hernia populations, more than half the patients had one or more lifestyle-related risk factor: nearly 40% had one risk factor and approximately 13% had two risk factors, whereas three or four risk factors were uncommon (*Fig. 2*). Obesity was the most frequent risk factor, present in approximately one-third of patients in each group.

### Patients with primary ventral hernias

Patients with and without lifestyle-related risk factors were of similar age, but there were more men in the group with lifestyle-related risk factors, which also had a slightly higher Charlson co-morbidity index (*Table 1* and *Table S1*). Most patients had undergone open repair, although laparoscopic repair and mesh use were more frequent among those with lifestyle-related risk factors. Defect width and follow-up duration (around 5.5 years) were similar in the groups with and without lifestyle-related risk factors. In the group with lifestyle-related risk factors, 71.3% of patients had one risk factor, 24.2% had two risk factors, 4.2% had three risk factors, and 0.3% had four risk factors.

**Table 1:** Characteristics of patients undergoing primary ventral or incisional hernia repair according to the presence of lifestyle related risk factors

	Primary ventral hernia		Incisional hernia	
	Any lifestyle-related risk factor (*n* = 8094)	No lifestyle-related risk factor (*n* = 7092)	Any lifestyle-related risk factor (*n* = 2094)	No lifestyle-related risk factor (*n* = 1679)
Age (years), median (i.q.r.)	53 (45–62)	52 (41–62)	58 (48–66)	61 (51–69)
**Sex**				
Male	6072 (75.0%)	4273 (60.3%)	976 (46.6%)	738 (44.0%)
Female	2022 (25.0%)	2819 (39.7%)	1118 (53.4%)	941 (56.0%)
**Married/registered partnership**	4704 (58.1%)	4566 (64.4%)	1173 (56.0%)	1039 (61.9%)
Data missing	49 (0.6%)	22 (0.3%)	9 (0.4%)	13 (0.8%)
**Charlson co-morbidity index**				
0	5905 (73.0%)	5571 (78.6%)	1029 (49.1%)	834 (49.7%)
1	1218 (15.0%)	828 (11.7%)	294 (14.0%)	223 (13.3%)
2	628 (7.8%)	448 (6.3%)	459 (21.9%)	384 (22.9%)
3	190 (2.3%)	121 (1.7%)	152 (7.3%)	122 (7.3%)
≥ 4	153 (1.9%)	124 (1.7%)	160 (7.6%)	116 (6.9%)
**Lifestyle-related risk factors**				
Current smoker	2155 (26.6%)	0 (0.0%)	601 (28.7%)	0 (0.0%)
BMI > 30 kg/m^2^	4688 (57.9%)	0 (0.0%)	1221 (58.3%)	0 (0.0%)
Weekly alcohol intake > 10 units	1847 (22.8%)	0 (0.0%)	318 (15.2%)	0 (0.0%)
Physical inactivity*	2103 (26.0%)	0 (0.0%)	643 (30.7%)	0 (0.0%)
**Surgical approach**				
Open	6134 (75.8%)	6076 (85.7%)	1082 (51.6%)	971 (57.8%)
Conventional laparoscopy	1656 (20.5%)	880 (12.4%)	815 (38.9%)	553 (32.9%)
Robotic laparoscopy	284 (3.5%)	123 (1.7%)	137 (6.5%)	99 (5.9%)
Converted	20 (0.2%)	13 (0.2%)	60 (2.9%)	56 (3.3%)
Use of mesh	6005 (74.2%)	4397 (62.0%)	1961 (93.6%)	1512 (90.1%)
**Mesh placement**				
Onlay	2608 (32.2%)	2255 (31.8%)	427 (20.4%)	374 (22.3%)
Retromuscular	449 (5.5%)	220 (3.1%)	497 (23.7%)	417 (24.8%)
Preperitoneal	1361 (16.8%)	1078 (15.2%)	238 (11.4%)	205 (12.2%)
IPOM	1550 (19.1%)	821 (11.6%)	789 (37.7%)	507 (30.2%)
Only suture	2089 (25.8%)	2695 (38.0%)	133 (6.4%)	167 (9.9%)
Unknown	37 (0.5%)	23 (0.3%)	10 (0.5%)	9 (0.5%)
Defect width (cm), median (i.q.r.)	1.5 (1.0–2.0)	1.0 (1.0–2.0)	3.0 (2.0–5.0)	3.0 (1.5–5.0)
Follow-up (months), median (i.q.r.)	69 (35–98)	66 (32–96)	71 (39–99)	66 (32–95)
**Overall recurrence**	1289 (15.9%)	1075 (15.2%)	611 (29.2%)	420 (25.0%)
Reoperation	266 (3.3%)	249 (3.5%)	153 (7.3%)	126 (7.5%)
Clinically confirmed†	222 (2.7%)	175 (2.5%)	129 (6.2%)	67 (4.0%)
Self-reported suspicion	801 (10.0%)	651 (9.2%)	329 (15.7%)	227 (13.5%)

Values are *n* (%) unless otherwise stated. *Physical inactivity was defined as being physically active for < 3.5 h/week. †Patient-reported as having been clinically confirmed by a medical doctor. i.q.r., interquartile range; BMI, body mass index; IPOM, intraperitoneal onlay mesh.

**Figure 3:**
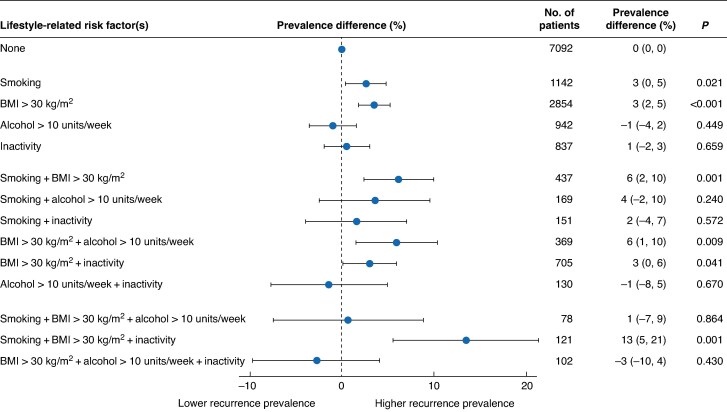
Forest plot of differences in the prevalence of recurrence after primary ventral hernia repair for different profiles of lifestyle-related risk factors Values in parentheses are 95% confidence intervals. The population-averaged recurrence prevalence in patients without lifestyle-related risk factors (reference group) was 14% (95% confidence interval 13% to 15%). Profiles based on group count < 50 are not shown. All prevalence differences were adjusted for age, sex, defect width, Charlson co-morbidity index, surgical approach, use of mesh, and the length of follow-up. Smoking was defined as daily smoking. Physical inactivity was defined as < 3.5 h of physical activity per week. BMI, body mass index.

In the main analysis, the adjusted prevalence of recurrence was 14.2% (95% c.i. 13.4% to 15.0%) in the scenario where the population had no lifestyle-related risk factors. On a population-average level, smoking only and obesity only, as well as the combination of the two, were associated with a significantly higher prevalence of recurrence (*Fig. 3*). In addition, the combinations of obesity with high alcohol intake, with physical inactivity, and with both smoking and physical inactivity were associated with a significantly higher prevalence of recurrence of an additional 3.0–13.4% (*Fig. 3*). The second analysis of the individual lifestyle factors showed that a higher prevalence of recurrence was specifically associated with current smoking and BMI 30–40 kg/m^2^ (*Table 2*). Unadjusted results are presented in *Tables S2* and *S3*, and adjusted prevalence rates are presented in *Table S4*.

**Table 2:** Impact of separate lifestyle-related risk factors on recurrence

	Primary ventral hernia			Incisional hernia		
	No. of patients	Difference in prevalence in percentage points	*P*	No. of patients	Difference in prevalence in percentage points	*P*
**Smoking status**						
Never smoked	9086	Reference		2074	Reference	
Ex-smoker	3945	1.3 (0.00, 2.7)	0.053	1098	3.8 (0.6, 7.1)	0.022
Current smoker	2155	3.3 (1.6, 5.0)	< 0.001	601	5.1 (0.9, 9.2)	0.016
**BMI (kg/m^2^)**						
< 18	58	−0.5 (−8.2, 7.2)	0.907	17	5.4 (−15.1, 25.9)	0.606
18–25	4110	Reference		968	Reference	
> 25–30	6330	0.7 (−0.7, 2.1)	0.313	1567	−0.3 (−3.8, 3.3)	0.879
> 30–35	3352	3.8 (2.1, 5.6)	< 0.001	843	2.0 (−2.1, 6.2)	0.330
> 35–40	988	4.8 (2.2, 7.5)	0.001	263	3.6 (−2.5, 9.8)	0.245
> 40	348	3.5 (−0.5, 7.5)	0.082	115	7.9 (−1.0, 16.7)	0.081
**Alcohol intake (units/week)**						
0–10	13 339	Reference		3455	Reference	
11–20	1535	−0.9 (−3.0, 1.1)	0.375	272	−5.3 (−10.7, 0.1)	0.052
> 20	312	0.1 (−4.3, 4.6)	0.954	46	0.1 (−13.0, 13.2)	0.992
**Physical inactivity**						
No	13 083	Reference		3130	Reference	
Yes	2103	0.0 (−1.6, 1.7)	0.988	643	0.0 (−3.8, 3.9)	0.981

Values in parentheses are 95% confidence intervals. For each lifestyle-related risk factor, the association with the prevalence of recurrence compared with the corresponding non-risky behaviour (reference) has been estimated as a difference in prevalence in percentage points. Estimates for each lifestyle factor were adjusted for the other three lifestyle factors as well as age, sex, defect width, Charlson co-morbidity index, surgical approach, use of mesh, and length of follow-up. BMI, body mass index.

Overall, the subgroup and sensitivity analyses were limited by small numbers of patients in many of the lifestyle groups, thereby reducing the statistical power and precision of the estimates. Subgroup analysis for patients with a defect size > 4 cm was not meaningful because the lifestyle-related risk factors subgroups were too small. Results from the group with a defect size < 4 cm were like those of the main analysis. Subgroup analysis of the open surgical approach did not show the same association between obesity and recurrence as observed in the main analysis. Otherwise, trends were similar but smaller in magnitude, and only the combination of smoking, obesity, and physical inactivity remained significant (data not shown). In contrast, for laparoscopic repair, smoking showed no association with recurrence, whereas obesity was the only lifestyle factor associated with a significantly higher recurrence prevalence (data not shown).

Sensitivity analysis restricted to patients with a follow-up duration < 5 years found only smoking to be associated with a significantly higher prevalence of recurrence in the primary ventral hernia group. The estimate for obesity was similar in magnitude to that in the main analysis (2.2% *versus* 3.5%, respectively), but with wider confidence intervals that included zero, and was therefore not statistically significant. This likely reflects reduced statistical precision due to the smaller sample size rather than the absence of an association. No trends were found for the combinations of two lifestyle factors, and all groups with combinations of > 2 factors had fewer than 50 patients and were not interpreted (*Table S5*). When restricting the outcome to reoperation for recurrence or clinically confirmed recurrence, the recurrence prevalence was 5.7% when the population had no lifestyle-related risk factors. Only associations with obesity independently (prevalence difference 1.2%; 95% c.i. 0.1 to 2.3; *P* = 0.036) remained significant (*Table S6*).

### Patients with incisional hernias

Age, sex, Charlson co-morbidity index, defect size, and follow-up (5.5–6 years) were similar between the groups with and without lifestyle-related risk factors (*Table 1*, and *Table S1*). The group with lifestyle-related risk factors had slightly more laparoscopic repairs and mesh use. In the group with lifestyle-related risk factors, 70.8% of patients had one risk factor, 25.7% had two risk factors, 3.4% had three risk factors, and 0.1% had four risk factors. In the main analysis, the adjusted prevalence of recurrence was 25.3% (95% c.i. 23.2% to 27.4%) for the population with no lifestyle-related risk factors.

**Figure 4:**
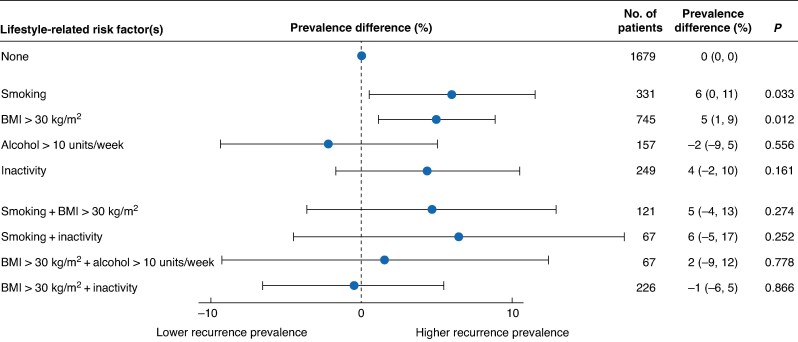
Forest plot of differences in the prevalence of recurrence after incisional hernia repair for different profiles of lifestyle-related risk factors Values in parentheses are 95% confidence intervals. The population-averaged recurrence prevalence in patients without lifestyle-related risk factors (reference group) was 25% (95% confidence interval 23–27). Profiles based on group count < 50 are not shown. All prevalence differences were adjusted for age, sex, defect width, Charlson co-morbidity index, surgical approach, use of mesh, and length of follow-up. Smoking was defined as daily smoking. Physical inactivity was defined as < 3.5 h of physical activity per week. BMI, body mass index.

The main analysis showed a significant association with a higher prevalence of recurrence for smoking only and obesity only, although the 95% c.i. values were wide (*Fig. 4*). A more detailed analysis of individual lifestyle factors showed a higher recurrence prevalence for both previous and current smoking, but not across BMI groups, alcohol intake, or physical inactivity (*Table 2*). The more detailed analysis showed that the difference in recurrence prevalence increased with increasing BMI; however, it was not significant in this analysis, and the confidence intervals were wide. Unadjusted results are presented in *Tables S2* and *S3*, and adjusted prevalences are presented in *Table S4*.

Again, the subgroup and sensitivity analyses were limited by the small number of patients in several of the lifestyle-related risk factor groups, which reduced the statistical power, and the 95% c.i. values were generally wide. In the subgroup with a defect size < 4 cm, only smoking was associated with a significantly higher prevalence of recurrence, as seen in the main analysis (data not shown). The association with obesity was also as found in the main analysis, but not significant. In the group with a defect size > 4 cm, only physical inactivity was associated with a significantly higher prevalence of recurrence, although this is based on only 71 patients (data not shown). Subgroup analysis based on surgical approach showed that only smoking was significantly associated with a higher recurrence prevalence in the open repair group, and only obesity was significantly associated with a higher recurrence prevalence in the laparoscopic group (data not shown).

In the sensitivity analysis with follow-up < 5 years, only the single lifestyle factor groups and the combination of obesity and physical inactivity had ≥ 50 patients. Among these factors, only smoking was associated with a significantly higher prevalence of recurrence (*Table S5*). The estimate for obesity was lower than in the main analysis (2.4% *versus* 4.9%) and not statistically significant, with wider confidence intervals crossing zero. However, the confidence intervals overlapped substantially with those of the main analysis, indicating that the observed difference is likely due to imprecision rather than a true absence of association. When restricting the outcome to reoperation for recurrence or clinically confirmed recurrence, the recurrence prevalence was 11.5% when the population had no lifestyle-related risk factors. Only associations with obesity as a single factor (prevalence difference 3.1%; 95% c.i. 0.2% to 6.0%; *P* = 0.035) remained significant (*Table S6*).

### Other analyses

A small proportion of patients experienced larger changes in BMI from the time of surgery to responding to the survey. In total, BMI decreased by > 5 kg/m^2^ in 3.8% of patients, and increased by > 5 kg/m^2^ in 3.6% of patients, and was within 5 kg/m^2^ in 92.6% of patients. The mean within-person difference in BMI was 0.00 kg/m^2^ (95% c.i. −0.07 to 0.07 kg/m^2^; *P* = 0.973), indicating no overall systematic change (*Fig. S1*).

## Discussion

In this nationwide 10-year cohort, more than half the patients undergoing primary ventral or incisional hernia repair had at least one lifestyle-related risk factor, most commonly obesity, affecting nearly one-third of patients. Overall, the estimated population-averaged recurrence prevalence was 14.2% after primary ventral hernia repair and 25.3% after incisional hernia repair among patients without lifestyle-related risk factors. The presence of one or more lifestyle-related risk factors increased the adjusted absolute recurrence prevalence to 16.8–27.5% after primary ventral hernia repair and to 30.3–31.3% after incisional hernia repair. For primary ventral hernia repair, current smoking and obesity (BMI > 30 kg/m^2^) were associated with a significantly higher prevalence of recurrence, both as isolated factors and when combined with other lifestyle-related risk factors. For incisional hernia repair, both current and former smoking were associated with a significantly higher prevalence of recurrence. Obesity showed a similar direction of association, although statistical significance was only observed in analyses with larger sample sizes.

When comparing these two ventral hernia populations with the general Danish population^32^, fewer patients reported alcohol consumption exceeding 10 units/week, particularly in the incisional hernia group. This could possibly be due to underreporting or secondary to co-morbid conditions. Second, obesity was considerably more prevalent in both hernia populations, with nearly one-third of patients classified as obese, compared with approximately one-fifth of the general Danish population of similar age^32^.

Previous studies on primary ventral hernia repair have reported inconsistent findings regarding the impact of smoking and obesity on recurrence. Most studies have identified obesity as a significant risk factor for recurrence^5,6,33–35^, although other studies reported conflicting results^36–38^. Similarly, evidence regarding smoking is inconsistent^39,40^. Furthermore, the magnitude of these associations remains uncertain due to heterogeneous study methods and varying effect measures, complicating direct comparisons across studies. In the present study, smoking and obesity were both associated with a higher risk of recurrence. In subgroup analyses, only obesity was associated with higher recurrence after laparoscopic repair, although the analysis was limited by small sample sizes. For open repair, no single lifestyle factor was significantly associated with recurrence, although the point estimate for smoking was similar to that in the main analysis. The significance of a high alcohol intake and the level of physical activity in relation to recurrence after ventral hernia repair is uncertain. The effects of high alcohol intake in relation to hernia repair have been very sparsely investigated, but an association to short-term postoperative complications has been shown across other surgical procedures^14,41^. Similarly, the effects of physical inactivity in ventral hernia repair have been sparsely investigated. One study^16^ found an association between low physical activity and short-term postoperative complications and readmission, but the role of physical activity in recurrence was less clear due to the short follow-up. The present study did not find a significant association between physical inactivity or high alcohol use and recurrence. The inverse association observed for alcohol intake > 10 units/week in unadjusted analyses is likely explained by confounding. Higher reported intake may reflect underlying socioeconomic and health-related factors associated with a lower baseline risk of recurrence. Adjustment for age, co-morbidity, and surgical characteristics, partly acting as proxies for these factors, markedly attenuated the association, indicating that the unadjusted finding was driven by baseline differences rather than a true protective effect. Misclassification may also have contributed, because former heavy drinkers classified as abstinent (‘sick quitter’ effect) and underreporting can bias estimates. Together, these factors likely explain the weak, non-significant inverse associations observed after adjustment.

A few studies^42,43^ have reported that smoking and obesity may interact synergistically, increasing the risk of postoperative complications in the short term beyond the effects of each factor alone, although one of these studies^43^ was based on a very small sample size. In the present study, no synergistic effect of concurrent smoking and obesity on recurrence was observed. The prevalence difference for this combination was 6.1%, indicating a possible additive effect; however, the 95% c.i. (2.4% to 9.9%) does not support a definitive conclusion. A small group of patients had the combination of smoking, obesity, and physical inactivity and had the highest prevalence difference of all of 13.4% (95% c.i. 5.5% to 21.2%). Despite the nationwide design and high response rate of this study, several subgroup analyses may have been underpowered due to small sample sizes in groups with multiple lifestyle-related risk factors. Therefore, additive effects cannot be excluded based on these results.

For incisional hernia repairs, smoking and obesity alone were associated with higher recurrence, although wide confidence intervals leave uncertainty about the true magnitude of these associations. Comparisons with existing literature are challenging due to the heterogeneous use of effect measures, recurrence definitions, and the frequent inclusion of incisional hernias in mixed cohorts. Still, both a large registry study^5^ and a systematic review^6^ with mixed hernia cohorts found the same trend of obesity being associated with increased recurrence risk, whereas the evidence for smoking remains inconsistent^5,6,39^. In the present study, smoking and obesity were both associated with a significantly higher prevalence of recurrence. However, on a population-average, recurrence would still occur in one in four patients under the counterfactual scenario of no lifestyle-related risk factors, indicating that factors other than lifestyle, such as surgical or patient-related characteristics, likely play an important role in recurrence after incisional hernia repair.

In the sensitivity analysis restricted to clinically confirmed recurrences (reoperation or self-reported clinically confirmed), fewer associations reached statistical significance than in the main analysis for both primary ventral and incisional hernias. Although obesity remained associated with recurrence, both as a single factor and, for the primary ventral hernias, in combination with smoking, effect estimates were smaller in absolute terms. This should be interpreted in the context of a substantially lower baseline recurrence prevalence (5.7% *versus* 14.2% for primary ventral hernias and 11.5% *versus* 25.3% for incisional hernias), which likely reduced absolute differences while the relative effects remained broadly similar. However, smoking alone was no longer significant in either hernia population. For combinations involving obesity and other lifestyle factors, effect estimates remained directionally consistent, but were no longer statistically significant, likely reflecting reduced statistical precision due to fewer events rather than the absence of an association. Overall, obesity and smoking appear to be associated with a higher recurrence prevalence for both primary and incisional ventral hernia repair, although the absolute effect size may be modest. Given the number of analyses performed, these findings should be interpreted with appropriate caution.

This study has several strengths. The data set comprises a nationwide cohort including all eligible patients over a decade, and with a very high response rate of 78%, which minimizes the risk of non-response bias that is often a limitation in survey studies. The nationwide coverage with inclusion of all private and all public hospitals generates a high external validity, and there is high validity of the operative data due to a high registration rate in Danish Ventral Hernia Database and high data accuracy^29,30^. Many studies likely underestimate recurrence due to small cohorts and short follow-up^44^, but in the present study the follow-up was long, and the sample sizes were large in most groups in the analyses of individual factors, increasing the reliability of results. Primary ventral and incisional hernias were analysed separately, as should be done^31^, despite this distinction often being overlooked^6^. Combining patient-reported recurrence and registry data may contribute to avoiding the underreporting of recurrence, especially among patients not seeking clinical examination. Comparisons with previously reported recurrence estimates are limited by heterogeneity in definitions, detection methods, and follow-up^5,6^. However, comparing prevalence in the present study with that in a large study from the USA with a 5-year follow-up^5^ and in another Danish study with follow-up up to 4 years^45^ using comparable definitions indicates that the recurrence estimates in this study are not overestimated. Finally, although there were notable changes in BMI in some individuals (∼7% had a change in BMI > 5 kg/m^2^ in either direction), there was no overall systematic shift between BMI at surgery and when patients completed the survey. This supports the use of current BMI as a reasonable proxy for BMI at the time of surgery, with only minor random error.

This study also has several limitations. First, there is an unavoidable selection bias, because around 5% of the Danish population does not use Digital Post^26^ and therefore did not receive the questionnaire on current lifestyle and experience of recurrence. Second, it is difficult to evaluate the impact of alcohol use due to frequent misclassification^46^ and unmeasured confounding, such as socioeconomic status^47^, impacting the results. Furthermore, self-reported intake often leads to underestimation^48^. In addition, the definition of high intake as > 10 units/week in this study may be lower than the level associated with postoperative complications in general^14,41^. Overall, this leads to limited reliability of the estimates of the association between weekly alcohol use and recurrence. Third, despite using a nationwide cohort with a high response rate, the small effect sizes and the limited number of patients in several lifestyle combination groups, especially in the incisional hernia cohort, likely resulted in underpowered analyses. Thus, the estimates for the combination groups are more prone to type I and II errors and appear less robust, as also reflected in the subgroup and sensitivity analyses. Fourth, a considerable proportion of recurrences (61.4% in the primary ventral group and 53.9% in the incisional group) were self-reported without clinical confirmation, risking misclassification and overestimation, because not all reported cases may represent true recurrence. The final limitation is the use of current lifestyle risk factor status instead of the status at the time of surgery. Because lifestyle may have changed since the time of surgery, this introduces potential misclassification of exposure, and the results may not fully reflect the effect of lifestyle at the time of surgery on recurrence risk. For example, a patient who quit smoking after surgery would be classified as a non-smoker despite having been a smoker at the time of surgery. Such misclassification is likely to be predominantly non-differential, because exposure was assessed uniformly for all patients in the survey and this would therefore tend to attenuate associations, leading to more conservative effect estimates. However, if lifestyle changes were influenced by postoperative outcomes, some degree of differential misclassification cannot be excluded. For BMI specifically, despite support for using current values as a proxy, some misclassification cannot be excluded. However, this is likely limited and predominantly non-differential and would also tend to attenuate associations rather than introduce systematic overestimation or underestimation.

Reducing the relatively high risk of recurrence after hernia repair is important to lessen the burden on both individual patients and society. Despite numerous observational studies on obesity and smoking, findings remain conflicting regarding the impact and prevalence of lifestyle-related risk factors and their association with recurrence. This study contributes new evidence to the field. Unfortunately, only few and small randomized clinical trials have evaluated preoperative lifestyle interventions in ventral hernia repair^49–52^, and these have primarily focused on 30-day postoperative complications. Therefore, it is difficult to build a strategy for reducing recurrence on solid evidence, and sizable randomized trials with superior or non-inferior designs are required. Using prevalence as the effect measure makes the findings easy to communicate in patient consultations. Still, recurrence represents only one outcome, and future studies should explore how these lifestyle factors affect other clinical and patient-reported outcomes to provide a more comprehensive understanding of their overall impact and to inform patient counselling, ventral hernia guidelines, and prehabilitation strategies.

## Supplementary Material

zrag098_Supplementary_Data

## Data Availability

Data sharing is not permitted due to Danish law.
