# Supplementary material for: Lifestyle-related risk factors and recurrence after ventral hernia surgery: nationwide registry and survey study
Source: BJS Open. 2026 Aug 11;10(4):zrag098. doi: 10.1093/bjsopen/zrag098 (PMC13459032; doi:10.1093/bjsopen/zrag098)
Supplement: Supplementary file 1 [file zrag098_supplementary_data.docx]

**Lifestyle-related risk factors and recurrence after ventral hernia surgery: Nationwide registry and survey study**

Sofie Anne-Marie Skovbo Jensen^1,2^, Siv Fonnes^1^, Anders Gram-Hanssen^1^, Hanne Tønnesen^2,3^, Hugin Reistrup^1^, Jacob Rosenberg^1,4^, Susanne Vahr Lauridsen^2,4^, Sara Nørgård Jensen^5^, Jason Joe Baker^1^

^1^ Center for Perioperative Optimization, Department of Surgery, Copenhagen University Hospital - Herlev and Gentofte, Borgmester Ib Juuls Vej 1, DK-2730 Herlev, Denmark

^2^ WHO-CC, the Parker Institute, Bispebjerg and Frederiksberg Hospital, DK-2000 Frederiksberg, Denmark

^3^ Department of Health Sciences, Faculty of Medicine, Lund University, Lund, Sweden

^4^ Department of Clinical Medicine, University of Copenhagen, Copenhagen, Blegdamsvej 3B, DK-2200 Copenhagen N, Denmark

^5^ Sanos, Søborg, Denmark

**Corresponding author.** Hanne Tønnesen **ORCID ID** 0000-0002-7161-3416

**Supplementary Materials - Index**

| **Supplementary Figures and Tables** |  |
| --- | --- |
| Supplementary Table 1 | *pag. 2* |
| Supplementary Table 2 | *pag. 3* |
| Supplementary Table 3 | *pag. 4* |
| Supplementary Table 4  Supplementary Table 5  Supplementary Table 6  Supplementary Figure 1 | *pag. 5*  *pag. 6*  *pag. 7*  *pag. 8* |

**Supplementary Tables**

**Supplementary Table 1.** **Detailed lifestyle characteristics matching analyses of combined SNAP factors.** “No lifestyle-related risk factors” is defined as never or previous smokers, BMI<30, weekly alcohol intake of 0–10 units, and physically active.

|  | **Characteristics** | | **Primary ventral hernia** | |  | **Incisional hernia** | |
| --- | --- | --- | --- | --- | --- | --- | --- |
|  |  |  | **No lifestyle-related risk factors**  n=7 092 | **Any lifestyle-related risk factors**  n=8 094 |  | **No lifestyle-related risk factors**  n=1 679 | **Any lifestyle-related risk factors**  n=2 094 |
|  | Smoking status, n (%) | |  |  |  |  |  |
|  |  | Never | 5 129 (72) | 3 957 (49) |  | 1 105 (66) | 969 (46) |
|  |  | Ex-smoker | 1 963 (28) | 1 982 (24) |  | 574 (34) | 524 (25) |
|  |  | Current | 0 (0) | 2 155 (27) |  | 0 (0) | 601 (29) |
|  | Nutrition, BMI, n (%) | |  |  |  |  |  |
|  |  | <18 | 32 (0) | 26 (0) |  | 3 (0) | 14 (1) |
|  |  | 18–25 | 2 946 (42) | 1 164 (14) |  | 625 (37) | 343 (16) |
|  |  | >25–30 | 4 114 (58) | 2 216 (27) |  | 1 051 (63) | 516 (25) |
|  |  | >30–35 | 0 (0) | 3 352 (41) |  | 0 (0) | 843 (40) |
|  |  | >35–40 | 0 (0) | 988 (12) |  | 0 (0) | 263 (13) |
|  |  | >40 | 0 (0) | 348 (4) |  | 0 (0) | 115 (5) |
|  | Alcohol intake, units/week, n (%) | |  |  |  |  |  |
|  |  | 0–10 | 7 092 (100) | 6 247 (77) |  | 1 679 (100) | 1 776 (85) |
|  |  | 11–20 | 0 (0) | 1 535 (19) |  | 0 (0) | 272 (13) |
|  |  | >20 | 0 (0) | 312 (4) |  | 0 (0) | 46 (2) |
|  | Physical inactivity, n (%) | | 0 (0) | 2 103 (26) |  | 0 (0) | 643 (31) |

All percentages are rounded to the nearest whole number. Physical inactivity is defined as being physically active <3.5 hours/week. BMI: body mass index, n: number.

**Supplementary Table 2. Unadjusted effects of different combinations of lifestyle-related risk factors on the prevalence differences for ventral hernia recurrence**

|  | | | | | **Primary ventral hernia** | | | | **Incisional hernia** | | | |
| --- | --- | --- | --- | --- | --- | --- | --- | --- | --- | --- | --- | --- |
| **Number of lifestyle-related risk factors** | | | | | **n** | **Diff. in prevalence to ref (95% CI)** | | **P-value** | **n** | **Diff. in prevalence to ref (95% CI)** | | **P-value** |
| 0 - reference | | | | | 7 092 | Ref |  | – | 1 679 | Ref |  | – |
| 1 | Smoking |  |  |  | 1 142 | 3% | (1–6) | 0.005 | 331 | 7% | (1–12) | 0.015 |
|  |  | BMI >30 |  |  | 2 854 | 1% | (-1–3) | 0.189 | 745 | 6% | (2–10) | 0.002 |
|  |  |  | Alcohol >10 |  | 942 | -5% | (-7–(-3)) | <0.001 | 157 | -5% | (-11–2) | 0.171 |
|  |  |  |  | Inactivity | 837 | 1% | (-2–3) | 0.710 | 249 | 4% | (-2–10) | 0.203 |
| 2 | Smoking | BMI >30 |  |  | 437 | 5% | (1–9) | 0.008 | 121 | 7% | (-1–16) | 0.099 |
|  | Smoking |  | Alcohol >10 |  | 169 | 0% | (-5–6) | 0.936 | 28 | -14% | (-26–(-3)) | 0.016 |
|  | Smoking |  |  | Inactivity | 151 | 3% | (-3–9) | 0.387 | 67 | 8% | (-4–19) | 0.180 |
|  |  | BMI >30 | Alcohol >10 |  | 369 | 0% | (-4–4) | 0.881 | 67 | -1% | (-12–9) | 0.831 |
|  |  | BMI >30 |  | Inactivity | 705 | 1% | (-1–4) | 0.326 | 226 | 1% | (-5–7) | 0.725 |
|  |  |  | Alcohol >10 | Inactivity | 130 | -5% | (-10–0) | 0.053 | 29 | -11% | (-24–2) | 0.084 |
| 3 | Smoking | BMI >30 | Alcohol >10 |  | 78 | -2% | (-10–5) | 0.539 | 2 | -25% | (-27–(-23)) | <0.001 |
|  | Smoking | BMI >30 |  | Inactivity | 121 | 12% | (4–20) | 0.003 | 37 | 13% | (-3–29) | 0.111 |
|  | Smoking |  | Alcohol >10 | Inactivity | 35 | 2% | (-11–15) | 0.756 | 12 | 8% | (-18–35) | 0.542 |
|  |  | BMI >30 | Alcohol >10 | Inactivity | 102 | -6% | (-12–(-1)) | 0.026 | 20 | 10% | (-11–31) | 0.352 |
| 4 | Smoking | BMI >30 | Alcohol >10 | Inactivity | 22 | -2% | (-16–13) | 0.836 | 3 | 8% | (-45–62) | 0.760 |

Smoking is defined as daily smoking, alcohol >10 is defined as a weekly intake >10 units, and inactivity is defined as physical activity <3.5 hours per week. CI: confidence interval, Diff: difference, n: number of patients, ref: reference.

**Supplementary Table 3. Unadjusted estimates of the impact of separate lifestyle-related risk factors on recurrence.**

|  | | **Primary ventral hernia** | | |  | **Incisional hernia** | | |
| --- | --- | --- | --- | --- | --- | --- | --- | --- |
|  | | n | Diff. in prevalence to ref (95% CI) | P-value |  | N | Diff. in prevalence to ref (95% CI) | P-value o |
| Smoking | |  |  |  |  |  |  |  |
|  | Never (ref) | 9 086 | – | – |  | 2 074 | – | – |
|  | Ex-smoker | 3 945 | 0% (-1–2) | 0.740 |  | 1 098 | 3% (0–6) | 0.076 |
|  | Current | 2 155 | 4% (2–6) | <0.001 |  | 601 | 6% (2–10) | 0.007 |
| Nutrition, BMI | | |  |  |  |  |  |  |
|  | <18 | 58 | 2% (-9–12) | 0.766 |  | 17 | 9% (-14–32) | 0.459 |
|  | 18–25 (ref) | 4 110 | – | – |  | 968 | – | – |
|  | >25–30 | 6 330 | -4% (-5–(-2)) | <0.001 |  | 1 567 | -1% (-5–2) | 0.485 |
|  | >30–35 | 3 352 | -1% (-3–0) | 0.085 |  | 843 | 2% (-2–6) | 0.330 |
|  | >35–40 | 988 | 1% (-2–3) | 0.558 |  | 263 | 5% (-1–12) | 0.099 |
|  | >40 | 348 | 2% (-2–6) | 0.404 |  | 115 | 11% (1–20) | 0.023 |
| Alcohol intake (units/week) | | | |  |  |  |  |  |
|  | 0–10 (ref) | 13 339 | – | – |  | 3455 | – | – |
|  | 11–20 | 1 535 | -4% (-6–(-3)) | <0.001 |  | 272 | -5% (-11–0) | 0.052 |
|  | >20 | 312 | -4% (-7–0) | 0.058 |  | 46 | 0% (-13–13) | 0.992 |
| Physical inactivity | | |  |  |  |  |  |  |
|  | No (ref) | 13 083 | – | – |  | 3 130 | – | – |
|  | Yes | 2 103 | 0% (-2–2) | 0.988 |  | 643 | 1% (-2–5) | 0.483 |

Physical inactivity is defined as physical activity <3.5 hours per week. Diff: difference, CI: confidence interval, N: total number of patients, ref: reference group.

**Supplementary Table 4. Adjusted prevalences of ventral hernia recurrence for different combinations of lifestyle-related risk factors.**

|  | | | | | **Primary ventral hernia** | | | **Incisional hernia** | | |
| --- | --- | --- | --- | --- | --- | --- | --- | --- | --- | --- |
| **Number of lifestyle-related risk factors** | | | | | **n** | **Prevalence (95% CI)** | | **n** | **Prevalence (95% CI)** | |
| 0 - reference | | | | | 7 092 | 14% | (13–15) | 1 679 | 25% | (23–27) |
| 1 | Smoking |  |  |  | 1 142 | 17% | (15–19) | 331 | 31% | (26–36) |
|  |  | BMI >30 |  |  | 2 854 | 18% | (16–19) | 745 | 30% | (27–34) |
|  |  |  | Alcohol >10 |  | 942 | 13% | (11–16) | 157 | 23% | (16–30) |
|  |  |  |  | Inactivity | 837 | 15% | (12–17) | 249 | 30% | (24–35) |
| 2 | Smoking | BMI >30 |  |  | 437 | 20% | (17–24) | 121 | 30% | (22–38) |
|  | Smoking |  | Alcohol >10 |  | 169 | 18% | (12–24) | 28 | 12% | (-1–25) |
|  | Smoking |  |  | Inactivity | 151 | 16% | (10–21) | 67 | 32% | (21–42) |
|  |  | BMI >30 | Alcohol >10 |  | 369 | 20% | (16–24) | 67 | 27% | (16–37) |
|  |  | BMI >30 |  | Inactivity | 705 | 17% | (14–20) | 226 | 25% | (19–30) |
|  |  |  | Alcohol >10 | Inactivity | 130 | 13% | (7–19) | 29 | 15% | (2–28) |
| 3 | Smoking | BMI >30 | Alcohol >10 |  | 78 | 15% | (7–23) | 2 | 0% | (0–0) |
|  | Smoking | BMI >30 |  | Inactivity | 121 | 28% | (20–35) | 37 | 36% | (21–50) |
|  | Smoking |  | Alcohol >10 | Inactivity | 35 | 20% | (6–34) | 12 | 32% | (6–57) |
|  |  | BMI >30 | Alcohol >10 | Inactivity | 102 | 11% | (5–18) | 20 | 36% | (15–57) |
| 4 | Smoking | BMI >30 | Alcohol >10 | Inactivity | 22 | 17% | (0–34) | 3 | 33% | (-14–80) |

All prevalences are rounded to the nearest whole number. Smoking is defined as daily smoking, alcohol >10 is defined as a weekly intake >10 units, and inactivity is defined as physical activity <3.5 hours per week. CI: confidence interval, n: number of patients.

**Supplementary Table 5. Sensitivity analysis on patients with follow-up <5 years**

|  | | | | | **Primary ventral hernia** | | | | **Incisional hernia** | | | |
| --- | --- | --- | --- | --- | --- | --- | --- | --- | --- | --- | --- | --- |
| **Number of lifestyle-related risk factors** | | | | | **n** | **Diff. in prevalence to ref (95% CI)** | | **P-value** | **n** | **Diff. in prevalence to ref (95% CI)** | | **P-value** |
| 0 - reference | | | | | 3217 | Ref |  | – | 752 | Ref |  | – |
| 1 | Smoking |  |  |  | 497 | 5% | (2–8) | 0.003 | 154 | 12% | (4–20) | 0.004 |
|  |  | BMI >30 |  |  | 1262 | 2% | (0–5) | 0.067 | 323 | 2% | (-3–8) | 0.392 |
|  |  |  | Alcohol >10 |  | 443 | -1% | (-5–2) | 0.420 | 60 | -8% | (-18–2) | 0.097 |
|  |  |  |  | Inactivity | 365 | -1% | (-4–3) | 0.680 | 104 | 2% | (-7–11) | 0.723 |
| 2 | Smoking | BMI >30 |  |  | 180 | 3% | (-2–9) | 0.204 | 42 | 8% | (-7–22) | 0.291 |
|  | Smoking |  | Alcohol >10 |  | 76 | 6% | (-3–15) | 0.190 | 11 | -23% | (-26–(-20)) | <0.001 |
|  | Smoking |  |  | Inactivity | 64 | 5% | (-3–13) | 0.238 | 26 | 0% | (-16–16) | 0.965 |
|  |  | BMI >30 | Alcohol >10 |  | 150 | 1% | (-5–8) | 0.668 | 34 | -13% | (-24–(-3)) | 0.012 |
|  |  | BMI >30 |  | Inactivity | 270 | 0% | (-4–4) | 0.930 | 73 | -5% | (-14–5) | 0.312 |
|  |  |  | Alcohol >10 | Inactivity | 52 | -4% | (-12–4) | 0.280 | 8 | -23% | (-26–(-20)) | <0.001 |
| 3 | Smoking | BMI >30 | Alcohol >10 |  | 31 | -3% | (-15–9) | 0.607 | 2 | -23% | (-26–(-20)) | <0.001 |
|  | Smoking | BMI >30 |  | Inactivity | 47 | 10% | (-2–23) | 0.003 | 13 | -8% | (-27–11) | 0.410 |
|  | Smoking |  | Alcohol >10 | Inactivity | 17 | 10% | (-11–31) | 0.067 | 6 | -9% | (-35–16) | 0.475 |
|  |  | BMI >30 | Alcohol >10 | Inactivity | 30 | -2% | (-15–10) | 0.420 | 10 | 18% | (-13–49) | 0.255 |
| 4 | Smoking | BMI >30 | Alcohol >10 | Inactivity | 13 | -9% | (-14–32) | 0.680 | 1 | -23% | (-26–(-20)) | <0.001 |

Smoking is defined as daily smoking, alcohol >10 is defined as a weekly intake >10 units, and inactivity is defined as physical activity <3.5 hours per week. CI: confidence interval, Diff: difference, n: number of patients, ref: reference.

**Supplementary Table 6. Adjusted sensitivity analysis defining recurrence as only reoperation or clinically confirmed**

|  | | | | | **Primary ventral hernia** | | | | **Incisional hernia** | | | |
| --- | --- | --- | --- | --- | --- | --- | --- | --- | --- | --- | --- | --- |
| **Number of lifestyle-related risk factors** | | | | | **n** | **Diff. in prevalence to ref (95% CI)** | | **P-value** | **n** | **Diff. in prevalence to ref (95% CI)** | | **P-value** |
| 0 - reference | | | | | 7 092 | Ref |  | – | 1 679 | Ref |  | – |
| 1 | Smoking |  |  |  | 1 142 | 0% | (-1–1) | 0.929 | 331 | 1% | (-3–5) | 0.727 |
|  |  | BMI >30 |  |  | 2 854 | 1% | (0–2) | 0.036 | 745 | 3% | (0–6) | 0.035 |
|  |  |  | Alcohol >10 |  | 942 | -1.5% | (-3–0) | 0.059 | 157 | 1% | (-4–7) | 0.606 |
|  |  |  |  | Inactivity | 837 | 1% | (-1–2) | 0.519 | 249 | 2% | (-3–6) | 0.451 |
| 2 | Smoking | BMI >30 |  |  | 437 | 3% | (0–5) | 0.039 | 121 | 5% | (-1.5–12) | 0.126 |
|  | Smoking |  | Alcohol >10 |  | 169 | -1% | (-4–2) | 0.544 | 28 | -11.5% | (-13–(-10)) | <0.001 |
|  | Smoking |  |  | Inactivity | 151 | -2% | (-5–1) | 0.304 | 67 | 3% | (-5–11) | 0.485 |
|  |  | BMI >30 | Alcohol >10 |  | 369 | 1% | (-2–4) | 0.476 | 67 | -3% | (-9–4) | 0.396 |
|  |  | BMI >30 |  | Inactivity | 705 | 2% | (0–4) | 0.084 | 226 | 2% | (-3–7) | 0.388 |
|  |  |  | Alcohol >10 | Inactivity | 130 | -3% | (-6–0) | 0.071 | 29 | -6% | (-14–2) | 0.160 |
| 3 | Smoking | BMI >30 | Alcohol >10 |  | 78 | -3% | (-7–1) | 0.136 | 2 | -11.5% | (-13–(-10)) | <0.001 |
|  | Smoking | BMI >30 |  | Inactivity | 121 | 3% | (-2–9) | 0.183 | 37 | 8% | (-4–20) | 0.200 |
|  | Smoking |  | Alcohol >10 | Inactivity | 35 | 4% | (-7–14) | 0.477 | 12 | 10% | (-12–32) | 0.380 |
|  |  | BMI >30 | Alcohol >10 | Inactivity | 102 | 0% | (-5–5) | 0.936 | 20 | -7% | (-16–2) | 0.142 |
| 4 | Smoking | BMI >30 | Alcohol >10 | Inactivity | 22 | 0% | (-11–10) | 0.975 | 3 | 21% | (-27–70) | 0.386 |

Smoking is defined as daily smoking, alcohol >10 is defined as a weekly intake >10 units, and inactivity is defined as physical activity <3.5 hours per week. CI: confidence interval, Diff: difference, n: number of patients, ref: reference.

**Supplementary Figure 1. Individual changes in BMI from surgery to survey response.**

BMI: body mass index.
